# Clinical Outcome of Femoral Suspensory Fixation for Anterior Cruciate Ligament (ACL) Reconstruction

**DOI:** 10.7759/cureus.78888

**Published:** 2025-02-12

**Authors:** Naif M Alhamam

**Affiliations:** 1 Orthopaedics, King Faisal University, Al-Ahsa, SAU

**Keywords:** anterior cruciate ligament injuries, cohort and prospective studies, follow-up studies, humans, knee

## Abstract

Objective

To assess the clinical and functional results of an anterior cruciate ligament (ACL) reconstruction performed by suspensory device fixation to the femur after an ACL rupture.

Methods

This is a prospective cohort study performed in Alahsa (eastern province of Saudi Arabia) on patients diagnosed with ACL injury who underwent femoral suspensory fixation for ACL reconstruction. Patients were clinically assessed for their knee functions and pain using the Oxford Knee Score (OKS) before and after the operation. These were then statistically analyzed to find out the changes in knee function, reduction in pain, and improvement of joint stability in general.

Results

Femoral suspensory fixation in ACL reconstruction yielded effective results on knee function, improvement, and pain reduction, thus supporting this technique as one of the reliable treatments for ACL injuries. It can also avoid further joint degeneration in the long run and keep the knees of active individuals healthy.

Conclusions

Femoral suspensory fixation in ACL reconstruction yielded effective results on knee function, improvement, and pain reduction, thus supporting this technique as one of the reliable treatments for ACL injuries. It can also avoid further joint degeneration in the long run and keep the knees of active individuals healthy.

## Introduction

The knee joint is a very complicated joint due to the number of ligaments regulating stability. Among these, the anterior cruciate ligament (ACL) is the most important for the stability of the knee and internal rotation. Therefore, ACL injuries are crucial and common injuries, comprising more than 50% of all knee injuries [1]. The ACL is one of the most important knee ligaments, arising from the posterior part of the medial surface of the lateral condyle of the femur and inserting into the anterior aspect of the intercondylar eminence of the tibia. It functions to prevent excessive anterior translation of the tibia in relation to the femur, while also providing rotational stability to the knee joint [2,3]. One of the most common and devastating injuries encountered by orthopedic surgeons is ACL rupture, mainly sustained by young active individuals during sports participation [4,5]. It usually occurs due to forceful hyperextension, twisting of the knee, or with a direct force applied to the lateral aspect of the knee either with a contact or non-contact mechanism [6, 7]. The estimated annual incidence of anterior cruciate ligament injury in the US ranges between 100,000 cases [8,9]. It is estimated that since 1980 more than 1.5 million ACL reconstructions were performed in the United States with estimated costs for treatment over three billion dollars annually [5]. Kim S et al. (2011) have declared that the number of ACL reconstructions performed has increased dramatically in recent years [10]. Dafalla S et al. (2020) emphasized that ACL injury is a common harm among the Saudi population, and its prevalence is 26.2% [11]. ACL rupture leads to short-term disability, puts an individual at high risk of future osteoarthritis [12]. Lohmander LS et al. (2007) highlighted that at 10 to 20 years after the ACL tear diagnosis, on average, 50% of diagnosed ACL tear patients have osteoarthritis with associated pain and functional impairment. ACL tears have shown to have negative physical and psychological impacts in the long term [13, 14]. Individuals with untreated ACL rupture are unable to resume high-level sport activities [15,16]. Furthermore, ACL rupture might lead to subsequent meniscal and articular cartilage injuries and worsening knee instability [17,18]. ACL injuries have shown to have negative consequences on athletes' careers, and it’s reported that athletes with ACL tears have functional, emotional, and financial consequences [19]. For that reason, in most cases, the decision among choices turns out to be an ACL repair [20,21]. The reconstruction of the ACL is important to keep the knee stable and to avoid osteoarthritic changes that can happen quite quickly. Clinical practice for ACL rupture management differs globally, and surgical techniques for ACL reconstruction have gone through many changes over the last two decades and have improved significantly. Currently, the mainstay of treatment for a torn ACL is an intra-articular reconstruction using a biologic graft [22,23]. The torn ACL is generally replaced surgically by a substitute graft made of tendon such as patellar tendon autograft, hamstring tendon autograft, quadriceps tendon autograft, and allografts [24]. The clinical success of the reconstruction of the ACL depends on many parameters, including graft material, graft fixation, graft placement, and post-reconstruction therapy. In cases of performing the ACL reconstruction, the results improve with the use of suspension fixation devices, which allow for an increase in the graft volume inside the femoral tunnel. Suspensory fixators have a button that rests on the thigh bone surface and a loop that maintains the folded ACL graft in place while it heals. They improve the success rate of ACL repair [25,26]. Based on searching on various databases like PubMed and Cochrane, no study has been conducted in Al-Ahsa, Saudi Arabia to measure clinical outcomes of ACL reconstruction using femoral suspensory fixation. Therefore, the purpose of this study is to identify patients diagnosed with ACL rupture who underwent femoral suspensory fixation of ACL reconstruction and to measure the clinical and functional outcomes post-operation.

## Materials and methods

Study design

This study utilized a retrospective cohort design to collect and analyze data from patients diagnosed with ACL rupture who underwent femoral suspensory fixation of ACL reconstruction and to measure the clinical and functional outcomes post-operation.

Study area and setting

The study was conducted in Alahsa (eastern province of Saudi Arabia). Inclusion criteria included patients with confirmed rupture of ACL through radiologic examination and clinical evaluation (e.g., MRI), patients 18 years and older, patients with femoral suspensory fixation included in the routine of ACL reconstruction, patients with full postoperative and preoperative clinical examination, including Oxford Knee Scores (OKS), and patients operated on in Al-Ahsa (eastern region of Saudi Arabia). Exclusion criteria included patients with concomitant severe pathologies in the knee (e.g., osteoarthritis, significant tear in the menisci necessitating individual surgical intervention), patients with alternative and adjunctive techniques of ACL reconstruction, patients with systemic disease affecting healing (e.g., autoimmune disease, coagulation disorder, diabetes mellitus), patients with incomplete follow-up and preoperative details such that analysis of clinical outcomes is not possible, patients with a past medical history of allergic reaction towards implanted materials in femoral suspensory implants, and patients with a past surgical and/or trauma history in the same knee not secondary to ACL rupture.

Sample size and sampling technique

The study aimed to review the medical records of 123 patients who met the inclusion and exclusion criteria. A retrospective chart review was performed on 82 patients aged 18 years or older diagnosed with ACL rupture confirmed by clinical examination and imaging, who were eligible to be included in this study.

Data management and statistical analysis

The data collected were edited, coded, and entered into statistical software IBM SPSS version 22 (SPSS, Inc., Chicago, IL). All computations in this study were done using two-tailed hypothesis tests where the alpha level was set at less than 0.05. It contains 12 questions: each question scores from 0 to 4. The total score is 48. Scores from 0 to 19 may indicate severe knee pain and require surgical intervention; 20 to 29 may indicate moderate to severe knee pain; 30 to 39 may indicate mild to moderate knee pain; and 40 to 48 may indicate satisfactory joint function. We included all the patients visiting orthopedic clinics who were diagnosed with ACL injury and underwent surgical fixation of ACL performed with the femoral suspensory fixation technique. We also contacted the official Oxford website to get permission to use the Oxford Clinical Knee Score in this study [27]. Descriptive analysis based on frequency and percent distribution was done for all variables, including patients’ personal data, body mass index, and their Oxford knee score distribution before and after femoral suspensory fixation of ACL reconstruction with overall knee arthritis grade. Significant changes in patients’ arthritis grades after surgery were tested using the McNemar test for paired data [28]. The distribution of patients' preoperative arthritis grades by their personal data and BMI was tested using the Pearson chi-square test and the exact probability test for small frequency distributions.

## Results

A total of 82 patients meeting the inclusion criteria were admitted (Table 1). Patients' ages ranged from 18 to 42 years, with a mean age of 29.9 ± 5.9 years. All patients were males. Exactly 34 (41.5%) patients had normal weights, 42 (51.2%) were overweight, and 6 (7.3%) were obese. Patients' weights ranged from 47 to 98 kg with a mean weight of 76.6 ± 9.9, and their heights ranged from 158 to 187 cm with a mean height of 172.9 ± 6.5 cm.

**Table 1:** Personal data of patients who underwent femoral suspensory fixation of ACL reconstruction. ACL: Anterior cruciate ligament.

Personal data	No.	%
Age in years		
18-24	21	25.6%
25-30	24	29.3%
31-35	22	26.8%
> 35	15	18.3%
BMI		
Normal weight	34	41.5%
Overweight	42	51.2%
Obese	6	7.3%
Height (cm)		
Range	158-187	Range
Mean ± SD	172.9 ± 6.5	Mean ± SD
Weight (Kg)		Weight (Kg)
Range	47-98	Range
Mean ± SD	76.6 ± 9.9	Mean ± SD
Range	158-187	Range
Mean ± SD	172.9 ± 6.5	Mean ± SD

OKS among patients who underwent femoral suspensory fixation of ACL reconstruction (Table 2). All OKS items showed significant improvement after ACL reconstruction surgery. Nearly all patients were in the none or slight effect categories, and none of them still had moderate to extreme difficulty. Also, 95.1% of the patients reported that they could walk down a flight of stairs easily after surgery compared to only 17.1% before undergoing the surgery (P = 0.001). Additionally, none of the patients had pain from the knee that interfered with their usual work after undergoing the surgery, versus only 13.4% before surgery (P = 0.001). All patients agreed that they could kneel down and get up again after the surgery, compared to 11% of them before surgery (P = 0.001). Additionally, all patients reported that they had no trouble getting in and out of the car or using public transport because of their knee after undergoing the surgery, compared to 9.8% before surgery (P = 0.001). Self-washing or drying was reported by all patients after undergoing the surgery compared to 7.3% before surgery (P = 0.001).

**Table 2:** Oxford Knee Score among patients who underwent femoral suspensory fixation of ACL reconstruction. P: McNemar test for related samples * P < 0.05 (significant) ACL: Anterior cruciate ligament.

Oxford knee score			Phase			
		Pre-operative		Post-operative		P-value
		No.	%	No.	%	
How would you describe the pain you usually have in your knee?	Severe	7	8.5%	0	0.0%	
	Moderate	35	42.7%	0	0.0%	
	Mild	34	41.5%	0	0.0%	
	Very mild	5	6.1%	3	3.7%	.001*
	None	1	1.2%	79	96.3%	
Have you had any trouble washing and drying yourself (all over) because of your knee?	Extreme difficulty	5	6.1%	0	0.0%	
	Moderate trouble	47	57.3%	0	0.0%	
	Very little trouble	24	29.3%	0	0.0%	.001*
	No trouble at all	6	7.3%	82	100.0%	
Have you had any trouble getting in and out of the car or using public transport because of your knee?	Extreme difficulty	8	9.8%	0	0.0%	
	Moderate trouble	40	48.8%	0	0.0%	
	Very little trouble	26	31.7%	0	0.0%	.001*
	No trouble at all	8	9.8%	82	100.0%	
For how long are you able to walk before the pain in your knee becomes severe?	Around the house only	6	7.3%	0	0.0%	
	5-15 minutes	13	15.9%	0	0.0%	
	16-60 minutes	59	72.0%	3	3.7%	.001*
	No pain > 60 min	4	4.9%	79	96.3%	
After a meal (sat at a table), how painful has it been for you to stand up from a chair because of your knee?	Very painful	10	12.2%	0	0.0%	
	Moderately pain	44	53.7%	0	0.0%	
	Slightly painful	23	28.0%	5	6.1%	.001*
	Not at all painful	5	6.1%	77	93.9%	
Have you been limping when walking because of your knee?	Most of the time	11	13.4%	0	0.0%	
	Often, not just at first	40	48.8%	0	0.0%	
	Sometimes or just at first	26	31.7%	2	2.4%	.001*
	Rarely / never	5	6.1%	80	97.6%	
Could you kneel down and get up again afterwards?	With extreme difficulty	2	2.4%	0	0.0%	
	With moderate difficulty	48	58.5%	0	0.0%	
	With little difficulty	23	28.0%	0	0.0%	.001*
	Yes, easily	9	11.0%	82	100.0%	
Are you troubled by pain in your knee at night in bed?	Most nights	5	6.1%	0	0.0%	
	Some nights	42	51.2%	0	0.0%	
	Only one or two nights	28	34.1%	11	13.4%	.001*
	Not at all	7	8.5%	71	86.6%	
How much has pain from your knee interfered with your usual work?	Greatly	5	6.1%	0	0.0%	
	Moderately	40	48.8%	0	0.0%	
	A little bit	26	31.7%	0	0.0%	001*
	Not at all	11	13.4%	82	100.0%	
Have you felt that your knee might suddenly give away or let you down?	Most of the time	73	89.0%	0	0.0%	
	Often, not just at first	8	9.8%	0	0.0%	
	Sometimes or just at first	0	0.0%	0	0.0%	.001*
	Rarely / never	1	1.2%	82	100.0%	
Could you do household shopping on your own?	With extreme difficulty	1	1.2%	0	0.0%	
	With moderate difficulty	22	26.8%	0	0.0%	
	With little difficulty	42	51.2%	0	0.0%	.001*
	Yes, easily	17	20.7%	82	100.0%	
Could you walk down a flight of stairs?	With moderate difficulty	44	53.7%	0	0.0%	
	With little difficulty	24	29.3%	4	4.9%	.001*
	Yes, easily	14	17.1%	78	95.1%	

**Figure 1:**
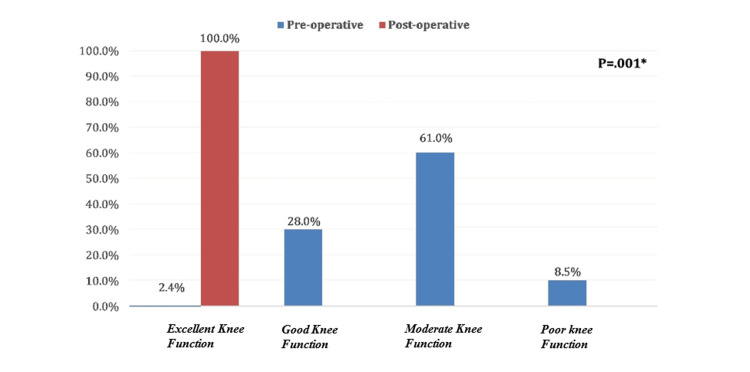
Overall Oxford Knee Score grade among patients who underwent femoral suspensory fixation of ACL reconstruction. ACL: Anterior cruciate ligament.

Overall OKS grade among patients who underwent femoral suspensory fixation of ACL reconstruction (Figure 1). Before surgery, only two (2.4%) patients had satisfactory joint function, 50 (61%) had moderate to severe arthritis, and 23 (28%) complained of mild to moderate arthritis, while all of them had satisfactory joint function after surgery with statistically significant change (P = 0.001).

Grade of knee arthritis severity among patients before surgery by their personal data. Exactly 66.7% of patients aged 18-24 years complained of moderate to severe arthritis compared to 73.3% of those aged above 35 years with no statistical significance (P = 0.139) (Table 3). Also, moderate to severe arthritis was detected among 55.9% of patients with normal weights versus all obese patients, with a statistically significant difference (P = 0.048).

**Table 3:** Grade of knee arthritis severity among patients before surgery by their personal data. P: Exact probability test *P < 0.05 (significant).

Grade of severity										
Personal data	Satisfactory joint function		Mild to moderate knee		Moderate to severe knee arthritis		Severe knee arthritis		P-value	
	No.	%	No.	%	No.	%	No.	%		
Age in years										
18-24	1	4.8%	6	28.6%	9	42.9%	5	23.8%		
25-30	1	4.2%	7	29.2%	16	66.7%	0	0.0%	0.139	
31-35	0	0.0%	6	27.3%	16	72.7%	0	0.0%		
> 35	0	0.0%	4	26.7%	9	60.0%	2	13.3%		
BMI										
Normal weight	2	5.9%	13	38.2%	17	50.0%	2	5.9%	0.048*	
Overweight	0	0.0%	10	23.8%	27	64.3%	5	11.9%		
Obese	0	0.0%	0	0.0%	6	100.0%	0	0.0%		

## Discussion

The present study was focused on evaluating the functional and clinical results after ACL reconstruction via femoral suspensory fixation in cases with a diagnosis of an injured ACL. As per the current study, relevant improvements in the function of the knee, pain, and stability of the joint postoperative intervention have been established through its output. A total of 82 patients participated in the study, and postoperative OKS showed significant improvement, with satisfactory function of the joint achieved in all the patients. As per the current observations, femoral suspensory fixation is an effective method for ACL reconstruction, with a definite improvement in knee stability and less pain. The study classified patients into four age groups. The first group ranged from 18 to 24 years, the second from 25 to 30 years, the third group from 31 to 35 years, and the last group included all patients above 35 years old. Almost a third of the patients belong to the second group, whereas less than 20% of the study population are more than 35 years old, likely due to the fact that ACL injury is mostly sustained by young individuals during sports participation. All patients in our study were males; this is attributed to the fact that females participate in sports much less than males in the area of study. The study showed an overall OKS grade among patients who underwent femoral suspensory fixation of ACL reconstruction before surgery that only 2 (2.4%) patients had satisfactory joint function, 50 (61%) had moderate joint function, and 23 (28%) had good joint function, while all of them had satisfactory with excellent joint function after surgery with statistically significant change (P=.001). This finding is consistent with a previous study addressing that Mean post-operative ACL reconstruction of International Knee Documentation Committee (IKDC) score and Lysholm score has been 75.6 and 84.4, respectively. Preinjury and post reconstruction mean Tegner scores were 5.4 and 4.26, respectively. Raymond et al. [29] emphasized that Postoperative Patient-Reported Outcomes Measurement Information System (PROMIS) physical function (PF), pain interference (PI), and depression (D) scores in patients who undergo ACL reconstruction showed significant improvement when compared with preoperative scores. According to our current study, 46.7% of patients aged 18 to 24 years complained of moderate joint function prior to ACL reconstruction surgery, compared to 73.3% of patients aged over 35 years, with no statistically significant difference (P=.139). Also, moderate joint function was detected among 55.9% of patients with normal weights versus all obese patients with a statistically significant difference (P=.048). That finding is in accordance with another finding pointed out that ACL injury most frequently happened in a younger population, and the ACL injury is linked to a 16% increased risk in developing knee OA compared to normal people [30]. It has been known that the ACL injury significantly leads to an increase in the inflammatory markers in the knee, which may influence the development of knee osteoarthritis [30]. Early ACL reconstruction led to the lowest incidence of degenerative changes on radiographic follow-up compared to delayed ACL reconstruction. There are a few limitations to this study that are important to highlight. First, the research was conducted at a single center in Alahsa, Saudi Arabia. This could limit how well the results apply to other populations. Further studies should include larger, more diverse populations to ensure that any findings could be considered to apply across diverse populations. This was, by design, a short-term follow-up study; very few cases followed patients longer than briefly. Longer-term follow-ups are needed, especially 5- and 10-year, which will show not only the durability of the femoral suspensory fixation but also any influence that may exist regarding long-term progression of knee arthritis. However, even though the present study demonstrated significant improvement in knee arthritis post-surgery, it remains unknown whether femoral suspensory fixation could prevent the onset of osteoarthritis once and for all. This study shall be continued to understand how different factors affect the development of knee OA after ACL reconstruction and what the efficacy of femoral suspensory fixation will be regarding the prevention of long-term degeneration of the knee. Moreover, it is also important to further investigate the relationship between BMI and knee arthritis progression, which can help inform management post-surgery.

## Conclusions

The findings of this study reveal that femoral suspensory fixation in ACL reconstruction is associated with significant improvements in patient-rated outcomes, particularly with regard to pain relief and knee function, as measured through the OKS. These improvements concur with previous studies, which have proposed that early ACL reconstruction can allow for functional restoration and possibly mitigate the development of long-term degenerative changes. Nevertheless, one must remember that our study concentrated predominantly on subjective outcomes, with objective stability values being withheld, and therefore, restricts one's ability to conclude definitively regarding the effectiveness of femoral suspensory fixation in re-establishing mechanical joint stability.

Although these observations add to the value of femoral suspensory fixation in ACL reconstruction, particularly in active persons, future studies with objective biomechanical assessments and long-term follow-up will be important in confirming its impact on the conservation of joints and osteoarthritis prophylaxis. In addition, future studies must evaluate factors such as age, weight, and preoperative state of joints in relation to surgical outcomes in an attempt to maximize selection criteria and therapeutic interventions for patients.
